# Host Glycan–Lectin Interplay in SARS-CoV-2 Infection

**DOI:** 10.3390/ijms27031608

**Published:** 2026-02-06

**Authors:** Hyeseong Oh, Vu Thi Thuy Tien, Showkot Ahmed, Jisoo Choi, Ki-Jun Ryu, Jinsung Yang

**Affiliations:** Department of Biochemistry and Convergence Medical Science, Institute of Medical Science, College of Medicine, Gyeongsang National University, Jinju 52727, Republic of Korea; hsoh2016@gmail.com (H.O.); thuytien.vu2501@gmail.com (V.T.T.T.);

**Keywords:** SARS-CoV-2, glycan-mediated viral entry, spike glycoprotein, host–virus interactions, lectins, heparan sulfate, sialic acid

## Abstract

Glycan-mediated processes can be critical determinants of viral attachment and entry, yet for enveloped RNA viruses, including SARS-CoV-2, their mechanistic roles remain incompletely defined. This review synthesizes current structural and functional evidence for glycan engagement during SARS-CoV-2 attachment and entry. We describe the general viral entry pathways and their reliance on glycan recognition, followed by the interactions of the SARS-CoV-2 spike glycoprotein with host glycans, including ABO(H) blood group antigens, sialylated glycans, and endogenous lectins. Based on structural biology, glycobiology, and virology, we focus on how the spike protein exploits both glycan motifs and lectin receptors to enhance attachment, promote cellular uptake, or modulate host tropism. We contextualize these mechanisms by comparing glycan dependencies across other human viruses, including the influenza virus, HIV, and norovirus. Finally, we provide a comparative virological perspective to derive broad evolutionary insights into how enveloped viruses exploit the host glycans.

## 1. Introduction

Glycans are complex carbohydrates covalently attached to proteins and lipids, and fundamental mediators of host–virus interactions. For many viruses, the earliest step of infection is enhanced by recognition of specific glycan motifs within the cell-surface glycocalyx, including sialylated structures, blood group antigens, and glycosaminoglycans such as heparan sulfate [1,2]. These interactions are often individually weak yet become biologically potent through multivalent display on virions and dense presentation on host membranes, thereby promoting viral retention at the cell surface and increasing the likelihood of productive engagement with high-affinity entry receptors.

A central class of glycan-interacting molecules involved in these processes is lectins. Lectins are carbohydrate-binding proteins that exhibit high specificity for specific sugar moieties within glycoconjugates and polysaccharides, often leading to the agglutination of cells or the precipitation of glycan-bearing macromolecules [3,4,5,6,7,8,9]. Through their ability to selectively recognize glycan structures, lectins play essential roles in cellular and molecular recognition events involving carbohydrates, proteins, and cells. Importantly, lectins also mediate the attachment and binding of bacteria, viruses, and fungi to their intended targets, positioning them as key modulators of host–pathogen interactions at mucosal and cellular interfaces [9].

Severe acute respiratory syndrome coronavirus 2 (SARS-CoV-2) uses its spike (S) glycoprotein to bind the angiotensin-converting enzyme 2 (ACE2) receptor, initiating viral entry [1]. However, growing evidences suggest that this interaction can be modulated by glycan-dependent contacts, including ABO(H) blood group antigens [1], sialic acid [10], and host lectins such as galectins and Siglecs [11,12,13]. These observations have extended the effect of glycan-mediated interactions to viral infectivity, tissue tropism, and susceptibility [1]. Studies suggest that glycan engagement may act as a priming or concentrating mechanism, enhancing ACE2-dependent binding [14].

Understanding glycan-mediated entry is critical not only for probing the early steps of SARS-CoV-2 infection but also for identifying broader principles shared with other viral pathogens such as influenza, HIV, and norovirus [15,16,17]. This review summarizes current knowledge on glycan recognition in SARS-CoV-2 entry, advancing our understanding of general mechanisms of glycan-dependent viral entry. We then compare these mechanisms with those employed by other human viruses to organize the evolutionary and functional diversity of glycan usage. The conclusion of this study is that glycan interactions, while variable in necessity, form a conserved strategy across many viral systems for host engagement and initiation of infection.

## 2. Virus Entry Mechanisms and the Role of Glycans

Viral entry into host cells is a highly coordinated, multistep process typically comprising: (1) initial attachment of the virion to the host cell surface, (2) engagement of a cognate entry receptor or co-receptor, and (3) membrane fusion, either directly at the plasma membrane or following endocytosis into endolysosomal compartments. While receptor binding and fusion have been extensively studied, the importance of the attachment phase, particularly glycan-mediated interactions, is increasingly recognized as a key determinant of infectivity and tropism [18,19].

The surfaces of vertebrate cells comprise a vast array of glycoconjugates, including N- and O-linked glycoproteins, glycosphingolipids, and proteoglycans. This glycan-rich matrix serves as both a defensive barrier and a molecular landscape that is suitable for exploitation by pathogens. Numerous viruses exploit cell-surface glycans as low-affinity but high-avidity attachment factors, enabling initial virion tethering and retention at the host cell interface. This initial binding dramatically increases the effective local concentration of virions at the membrane, thereby enhancing the likelihood of subsequent engagement with high-affinity entry receptors and triggering conformational changes in viral fusion proteins [20,21].

Glycans play diverse roles across a wide range of viruses. In some viruses, such as orthomyxoviruses, adenoviruses, and paramyxoviruses, specific glycans serve as the primary receptor. Influenza A virus hemagglutinin (HA), for instance, directly binds terminal sialic acid residues, with linkage-specific recognition (α2,3, α2,6) influencing host range and tissue tropism [10,22,23]. In other cases, glycans serve as accessory attachment factors. Heparan sulfate proteoglycans (HSPGs), for example, are used by herpesviruses, papillomaviruses, flaviviruses, and SARS-CoV-2 to enhance viral adherence and initiate conformational rearrangements of fusion machinery [14,24]. Mucin-type O-glycans and sulfated glycans likewise function in localizing viral particles to mucosal surfaces, while lectin-expressing cells such as dendritic cells and macrophages facilitate trans-infection by capturing virions via glycan-binding receptors such as DC-SIGN (CD209) and Siglec-1 (CD169) [25,26].

These interactions between the virus and glycan are generally of low monovalent affinity, often with a dissociation constant (*K_D_*) in the millimolar range [27]. However, viruses overcome this limitation through multivalency. Influenza virions, for example, bear ~300 HA trimers, each of which can bind three sialic acid residues. This geometric and topological multivalency allows simultaneous engagement of dozens to hundreds of glycan ligands across the cell surface, resulting in strong adhesion and triggering downstream entry processes [10]. Similarly, the SARS-CoV-2 spike is a heavily glycosylated trimer, and recent reports propose multiple sites capable of glycan engagement, potentially enabling interactions with cell-surface glycans in cis (on the same membrane) or trans (across opposing surfaces) [15,28,29]. On the host side, lectins such as galectins, which form lattice-like oligomers, and siglecs, which can cluster within membrane microdomains, further amplify avidity-based retention and bridging of virions to target cells [29,30].

Glycan conformational plasticity arises from rotatable glycosidic linkages, branching, and postsynthetic modifications such as sulfation or acetylation, which increase the complexity of these interactions. Viral glycan-binding proteins often exhibit some tolerance to structural heterogeneity, enabling them to bind related glycoepitopes across tissues or species. However, subtle differences in glycan specificity, such as selectivity for α2,6 versus α2,3 sialic acid linkages or for 9-O-acetylated sialic acids, can be key determinants of host tropism, pathogenicity, and zoonotic potential [10,22,23].

Within this framework, SARS-CoV-2 presents a compelling model for examining how glycan interactions modulate viral entry. While ACE2 is the primary entry receptor, the role of additional glycan-mediated contacts in enhancing, stabilizing, or localizing spike-ACE2 engagement is now a focus of intense structural and biochemical investigation [13,22,23,28]. In the following sections, we delineate the canonical spike-ACE2 entry mechanism, review the evidence for direct and indirect glycan interactions, and explore how multivalent lectin-mediated processes may fine-tune the early stages of SARS-CoV-2 infection.

## 3. SARS-CoV-2 Entry and Glycan Interactions

**Figure 1:**
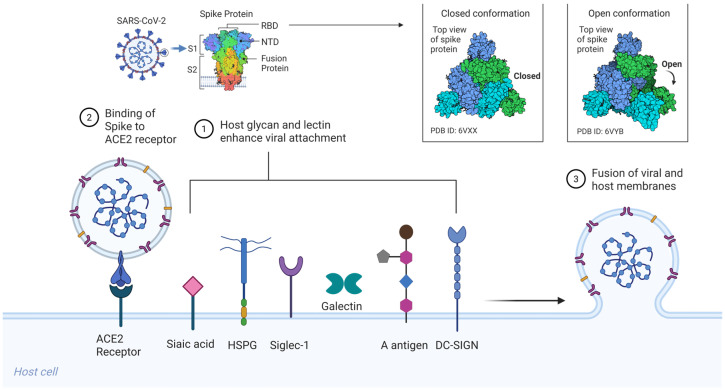
Host glycan and lectin-mediated SARS-CoV-2 entry. Sialylated glycans and glycolipids, including gangliosides (GM1, GM2, GM3) and mucin-associated sialoglycans, serve as low-affinity but multivalent attachment factors that engage the spike via the RBD and NTD. HSPGs on the host cell surface bind to the SARS-CoV-2 spike through electrostatic interactions involving a basic patch in the RBD and the S1/S2 PRRAR, thereby immobilizing virions and enhancing ACE2 encounter. Siglec-1, expressed on dendritic cells and macrophages, captures virions through host-derived sialylated gangliosides incorporated into the viral envelope, enabling the trans-infection of ACE2-positive target cells. Soluble galectins (Gal-3, Gal-9) and membrane-bound C-type lectin receptors (DC-SIGN, L-SIGN, MR) bind spike N-glycans, promoting virion retention, cross-linking, and immune cell-mediated dissemination. ABO(H) blood group antigens expressed on mucosal epithelial surfaces modulate spike attachment, with preferential recognition of blood group A trisaccharides and minimal binding to the unmodified H(O) antigen. Together, these interactions form a multivalent glycan–lectin network that enhances viral attachment and cell-associated spread prior to ACE2-dependent membrane fusion. Created in BioRender. Oh, H. (2026) https://BioRender.com/59lcolt, accessed on 3 February 2026.

SARS-CoV-2, like SARS-CoV (2003), initiates infection via the trimeric S glycoprotein, a class I viral fusion protein that drives receptor engagement and membrane fusion [21]. Each spike protomer (~1273 amino acids) comprises an S1 subunit, which includes the receptor-binding domain (RBD), and an S2 subunit that houses the fusion peptide, heptad repeat regions (HR1/HR2), transmembrane domain, and cytoplasmic tail [Figure 1] [20,21,24]. The S1 RBD directly binds the host receptor ACE2, while the S2 subunit mediates membrane merger following activation. Entry requires two proteolytic cleavages: one at the S1/S2 boundary and a second at the S2′ site, typically catalyzed by cell surface proteases such as TMPRSS2 or endosomal cathepsins, depending on the cellular context [20,22].

Structurally, spike trimers exist in a dynamic ensemble of conformations. Cryo-electron microscopy (cryo-EM) has revealed that the RBD transitions between a down (receptor-inaccessible) and an up (receptor-competent) state [20,21]. The down conformation helps shield neutralizing epitopes, while the up conformation is required for ACE2 binding. It has been proposed that initial low-affinity interactions, particularly with cell-surface glycans, may stabilize the RBD in the up conformation or promote lateral clustering of spike trimers, thereby enhancing receptor engagement and viral entry [23,24,28].

### 3.1. The Glycan Coat of Spike: Structural Landscape and Functional Implications

SARS-CoV-2 spike is heavily glycosylated, with each protomer bearing 22 N-linked glycan sites and several putative O-glycosylation motifs [20,21]. This glycan shield comprises approximately 40% of the molecular surface area [31], serving canonical roles in immune evasion by masking antigenic epitopes. Mass spectrometry-based glycoproteomic profiling has shown that these glycans are highly heterogeneous: sites on the S2 domain predominantly carry oligomannose-type structures, while those on S1 are typically processed into hybrid or complex-type glycans [21]. These site-specific glycoforms extend beyond structural roles and can directly influence spike conformational dynamics and receptor accessibility.

In particular, the N-glycans at N165 and N234 within S1 have been shown, through molecular dynamics simulations and cryo-EM modeling, to stabilize the RBD in the up position by engaging in intramolecular contacts with nearby residues [31]. This allosteric role suggests that spike glycans are not passive shields but functional regulators of conformational transitions critical to infectivity. Additionally, the RBD itself carries two N-glycans (N331 and N343) that are solvent-exposed and positioned near the ACE2 interface. NMR and glycan array studies have demonstrated that these glycans can engage macrophage galactose-binding lectin (MGL, CLEC10A), a C-type lectin receptor expressed on myeloid cells [20]. Although MGL typically recognizes GalNAc termini on O-glycans, it appears that certain processed spike N-glycans may present compatible epitopes, potentially facilitating lectin-mediated attachment to antigen-presenting cells or macrophages.

### 3.2. Host Glycans as Spike Attachment Factors

Beyond spike’s intrinsic glycosylation, several classes of host cell surface glycans function as attachment factors that can enhance SARS-CoV-2 entry. These include glycosaminoglycans, particularly heparan sulfate, and sialylated glycans displayed on glycoproteins, glycolipids, and mucins [23,24,28].

HSPGs are highly sulfated, linear polysaccharides that are anchored to the cell surface. Early in the pandemic, several independent studies revealed that SARS-CoV-2 spike binds heparan sulfate with moderate affinity and that enzymatic degradation of HSPGs or genetic knockout of HS biosynthesis enzymes significantly attenuates viral entry [14,24]. Mechanistically, a basic patch on the RBD, along with the S1/S2 polybasic cleavage site (PRRAR), engages negatively charged sulfate groups on HS chains, likely immobilizing the virion and promoting receptor encounter. Computational docking, site-directed mutagenesis, and biophysical binding assays support a model in which HS acts as a low-affinity but multivalent co-receptor that allosterically modulates spike conformation and enhances ACE2 interaction [20,29,31].

Sialylated glycans, terminal sialic acid-containing structures commonly found on mucosal surfaces and gangliosides, have also been implicated in viral attachment. Although early glycan array assays using isolated RBD yielded inconsistent results, more sensitive platforms such as native mass spectrometry and single-molecule force spectroscopy have reported interactions consistent with spike engagement of sialylated ligands. Reported targets include ganglioside-type sialosides (GM1, GM3), potentially via the RBD, and 9-O-acetylated sialic acid-modified glycoproteins, potentially via the N-terminal domain (NTD) [22,23,31,32]. These interactions exhibit modest affinity (*K_D_* ~100–200 µM), but the multivalent display on virions and the glycan-rich nature of the airway epithelium likely confer biologically relevant avidity.

In sum, available evidence supports a model in which SARS-CoV-2 entry is facilitated by sequential glycan and receptor interactions. Initial attachment to heparan sulfate and, in some contexts, sialylated glycans can concentrate virions at the cell surface, thereby increasing the likelihood of ACE2 engagement, after which proteolytic priming enables fusion. The presence of multiple glycan-binding modalities, involving both the RBD and NTD, emphasizes a nuanced role for glycan interactions in shaping viral infectivity.

## 4. Glycans and Lectins Participate in SARS-CoV-2 Entry

### 4.1. Sialylated Glycans and Siglecs

Sialic acids (Neu5Ac and its derivatives) are terminal residues on a wide range of cell-surface glycoproteins and glycolipids, particularly in the respiratory and gastrointestinal epithelia [33,34]. Their negative charge and strategic membrane localization make them prime targets for pathogen engagement. For SARS-CoV-2, emerging evidence highlights a multifaceted role for sialylated glycans in facilitating viral adhesion and possibly modulating tropism [20,32]. Biochemical and biophysical studies reveal that the Spike RBD binds to sialylated glycans, specifically α2,3 and α2,6 sialylated lactosamines, albeit with modest affinity (in the millimolar range) [20,32]. However, this binding is significantly potentiated when these glycans are presented in the context of gangliosides such as GM1 and GM2. Native mass spectrometry confirmed the formation of RBD-ganglioside complexes, while liposomal reconstitution systems demonstrated the recruitment of spike to membranes harboring these glycolipids. These results highlight the crucial role of lipid-anchored glycan presentation in enhancing spike binding [28,32].

At the cellular level, the enzymatic removal of surface sialic acids by neuraminidase diminishes pseudoviral entry, and glycoengineered cells overexpressing sialyltransferases exhibit increased susceptibility to viral infection. Genetic ablation of sialylation pathways, as shown in modified COS-7 cells, further supports a direct functional role of sialic acids in mediating Spike-dependent viral entry [32,35].

The spike NTD has emerged as a putative sialoside-binding module. In lineage A coronaviruses, such as OC43 and HKU1, a conserved sialic acid-binding pocket in the NTD accommodates 9-O-acetylated Neu5Ac [36]. Structural comparisons suggest that SARS-CoV-2′s NTD possesses a structurally flattened but potentially permissive pocket involving residues ~140–150 and ~170–180 [37]. Computational docking and biochemical assays indicate that variants such as Beta (B.1.351), which harbor NTD mutations like ins214EPE, may exhibit enhanced sialyl-glycan recognition. This raises the possibility that SARS-CoV-2 has latent capacity to evolve higher sialic acid avidity, with implications for tropism and immune evasion [37,38].

Functionally, sialylated glycans may serve as ephemeral tethering points that hold virions in proximity to mucosal surfaces. Mucins, rich in O-linked sialoglycans, can act as both barriers and viral concentrators [20,32]. On the apical membrane, ganglioside-rich lipid rafts, which often colocalize with ACE2, may spatially organize viral binding and entry [20,32]. Furthermore, an intriguing hypothesis posits that glycan engagement, particularly via the NTD, may allosterically shift Spike toward receptor-accessible conformations, thereby priming ACE2 interaction [32].

Siglecs (sialic acid-binding immunoglobulin-type lectins) constitute a family of host glycan-recognizing receptors, several of which are expressed on immune cells [20]. Siglec-1 (CD169) is particularly notable: it binds α2,3 sialylated gangliosides, including GM3, and is prominently expressed on dendritic cells and macrophages [25,39]. Pathogens such as HIV-1 and Ebola virus exploit Siglec-1 to adhere to and be transported by antigen-presenting cells in a trans manner [25,39,40]. SARS-CoV-2 appears to use a similar route. Enveloped virions incorporate host-derived gangliosides in their membrane and can be captured by Siglec-1 (CD169) on monocyte-derived dendritic cells, which can then mediate trans-infection of ACE2-positive target cells. Antibody-mediated blockade of Siglec-1 impairs this transfer, and ultrastructural analyses have visualized virions entrapped in Siglec-1-rich compartments [39].

This interaction does not appear to directly mediate productive infection of macrophages, but rather facilitates trans-infection and may amplify cytokine production. Siglec-1 engagement induces inflammatory signaling cascades that could contribute to immunopathology. The interaction relies on multivalent sialylated ligands, consistent with the densely glycosylated nature of the virion envelope and spike trimer [39,40]. While the spike N-glycans may contribute, viral membrane-incorporated gangliosides likely represent the dominant Siglec-1 ligands [39,41,42].

The exploitation of sialylated glycans by SARS-CoV-2 spans both epithelial attachment and immune cell engagement [20,32,39]. Putative glycan-interacting sites within the spike NTD and RBD, together with incorporation of host-derived glycolipids into the viral envelope, can support a multivalent interaction landscape [20,32]. Accordingly, interventions that disrupt these contacts, including sialic acid mimetics, Siglec-1 targeting approaches, or ganglioside-based decoys, represent potential adjunct strategies to reduce cell-associated viral spread and downstream immune activation [28,32,39].

### 4.2. Galectins and Lectins

Galectins are a family of soluble β-galactoside-binding lectins that are released to the extracellular space where they can cross-link glycoproteins on the cell surface and within the extracellular matrix [34,43,44]. Galectins are receiving increasing attention in SARS-CoV-2 research because spike contains a galectin-like domain implicated in glycan binding and because host galectins may influence viral attachment, fusion, and immune responses [2,43].

As previously noted, the RBD of SARS-CoV-2 bears significant structural similarity to galectin carbohydrate recognition domains (CRDs), notably galectin-3 (Gal-3) and galectin-4 [2,43]. Despite relatively low sequence identity (~11%), the RBD’s β-sandwich fold mimics the galectin scaffold, and surface electrostatic similarities further support functional convergence [2,34]. This proposed structural mimicry could enable the RBD to engage glycan motifs that are typically recognized by galectins. Indeed, Ryzhikov et al. reported that SARS-CoV-2 Spike binds poly-N-acetyllactosamine (LacNAc) chains after desialylation, exposing Galβ1-4GlcNAc units, the canonical ligands for galectin-3 and galectin-9 (Gal-9) [15]. It has been proposed that co-infection with neuraminidase-expressing pathogens, such as influenza virus, could enhance SARS-CoV-2 binding by removing terminal sialic acids and exposing underlying LacNAc (N-acetyllactosamine) motifs [18]. While the in vivo relevance remains speculative, this model suggests that the Spike RBD can exploit glycan remodeling events in the airway to gain enhanced adhesion.

On the host side, galectins themselves participate in viral interactions. Galectins can mediate immune signaling, modulate inflammation, and directly interact with viral glycoproteins. For SARS-CoV-2, Gal-3 and Gal-9 are particularly implicated:

Galectin-3: Widely expressed and upregulated in inflamed pulmonary tissues, Gal-3 possesses a single CRD and an N-terminal domain enabling oligomerization [43,44]. It binds poly-LacNAc motifs frequently found on complex-type N-glycans, including many on the Spike protein. Gal-3 has been shown to bind SARS-CoV-2 Spike S1 subunit and may facilitate virion aggregation or cross-linking to host cell glycans [45]. Functionally, Gal-3 binding appears to modulate infectivity. One study suggested Gal-3 stabilizes the prefusion conformation of Spike, potentially enhancing fusion competency [41,45]. Conversely, pharmacological inhibition of Gal-3 using small molecules or competing carbohydrates has shown partial reduction in viral entry in vitro, supporting a pro-viral role for Gal-3 under some conditions [41]. Notably, elevated Gal-3 levels are observed in the lungs and plasma of patients with severe COVID-19, suggesting that inflammation-induced Gal-3 may contribute to a positive feedback loop that exacerbates the infection [41,46].

Galectin-9: A tandem-repeat galectin with two CRDs, Gal-9 is known for its immunoregulatory properties, including modulation of T cell exhaustion via TIM-3 ligation. Gal-9 was recently shown to potently enhance SARS-CoV-2 attachment and entry in a glycan-dependent fashion [47]. When added exogenously to ACE2-expressing cells, Gal-9 augmented pseudovirus infection; this effect was abrogated by glycosidase treatment, confirming the necessity of glycan interactions [8]. Mechanistically, Gal-9 likely acts as a molecular bridge, simultaneously binding glycans on the Spike protein (e.g., terminal Galβ1-4GlcNAc on N-glycans) and on host cell surfaces, thereby increasing virion docking. This multivalency-mediated cross-linking mirrors the role of extracellular matrix lectins and may increase the local concentration of virions near receptor-rich zones.

These findings illustrate a recurring theme in viral glycobiology in which viruses may co-opt endogenous glycan-binding proteins (lectins) to support initial adhesion and potentially to influence immunomodulatory or structural pathways [43,44]. Variability in galectin expression across individuals, shaped by age, fibrosis, diabetes, and inflammation, could therefore contribute to differential susceptibility.

Beyond galectins, membrane-bound C-type lectin receptors also contribute to SARS-CoV-2 entry and dissemination. L-SIGN (CD209L) and DC-SIGN (CD209), expressed on pulmonary endothelial cells and dendritic cells, respectively, bind high-mannose and fucosylated N-glycans through calcium-dependent CRDs [21,26,48]. Experimental overexpression of DC/L-SIGN in receptor-deficient cells enables SARS-CoV-2 pseudovirus entry in the absence of ACE2, highlighting its role as an alternative attachment factor [26,48]. The Spike protein possesses multiple high-mannose N-glycans (notably at N234, N709, N717) that serve as ligands for these lectins [21]. Multivalent binding by tetrameric SIGN-family receptors results in high-avidity attachment, enhancing retention and trans-infection. in vivo, L-SIGN expression in liver and lung endothelial beds raises the possibility of viral dissemination through these compartments, while DC-SIGN-mediated capture by dendritic cells may facilitate immune cell-mediated virus transfer [21,26,48].

Similarly, the mannose receptor (MR, CD206), expressed on macrophages, binds terminal mannose, fucose, or GlcNAc residues and has been shown to capture Spike glycoprotein and mediate its internalization [41,49,50]. While MR-mediated entry is generally non-productive, it may contribute to antigen presentation and innate immune sensing, thereby promoting antiviral responses in early infection [49,51]. However, excessive MR-dependent uptake and signaling in macrophages may also amplify pro-inflammatory cytokine production and contribute to immunopathology in severe disease [52,53]. Thus, MR-mediated immune activation can exert either protective or detrimental effects on clinical recovery, depending on the timing, magnitude, and inflammatory context [54,55].

In conclusion, the host lectin landscape, comprising secreted galectins and membrane-bound C-type lectins, interacts dynamically with the glycan-rich surface of the SARS-CoV-2 virion. These interactions expand the canonical ACE2-centric model of entry, highlighting auxiliary pathways of virion capture, adhesion, and immune engagement. Far from being passive sugar decorations, the viral and host glycomes function as active determinants of infectivity and pathogenesis. Understanding this expanded glycan–lectin interactome provides a critical framework for dissecting viral tropism and identifying new intervention points in the SARS-CoV-2 infection cycle.

### 4.3. ABO(H) Blood Group Antigens

One of the most notable epidemiological observations early in the COVID-19 pandemic was the correlation between ABO blood type and susceptibility to SARS-CoV-2. Multiple independent studies have reported that individuals with blood group O exhibit a modestly reduced risk of infection, whereas those with blood group A are disproportionately represented among infected cohorts [10,56,57]. Although confounding factors and methodological variability were debated, a consistent trend emerged, echoing patterns observed during the SARS-CoV outbreak, where type O individuals were similarly underrepresented among severe cases [10,58]. A prevailing hypothesis proposed that natural anti-A and anti-B antibodies might neutralize virions carrying ABO(H) antigens acquired from host cell membranes. However, a 2021 report suggested a molecular mechanism by showing that the SARS-CoV-2 spike protein can engage ABO(H) blood group antigens on respiratory epithelial cells [2].

ABO(H) antigens are terminal carbohydrate epitopes built upon the H antigen core (Fucα1-2Galβ1-3/4GlcNAcβ-), which is further decorated with N-acetylgalactosamine (GalNAc) to yield A antigen or galactose to yield B antigen [59,60]. On erythrocytes, ABO(H) antigens are presented mainly on type 2 chains with Galβ1-4GlcNAc termini. In contrast, on secreted mucins and many epithelial surfaces, they are frequently carried on type 1 chains with Galβ1-3GlcNAc termini, with expression strongly influenced by FUT2 secretor status [33,34,59,61]. Critically, mucosal epithelial cells in the respiratory and gastrointestinal tracts express type I ABO(H) structures. Wu et al. demonstrated that the SARS-CoV-2 RBD preferentially recognizes the type I blood group A trisaccharide (GalNAcα1-3(Fucα1-2)Galβ-) over its type II counterpart [2]. Glycan microarray screening revealed robust binding to A(type I), weaker binding to A(type II) and B antigens, and negligible binding to the unmodified H(O) antigen [2]. Notably, the RBD exhibited little affinity for ABO determinants as expressed on red blood cells but a clear preference for the mucosa-associated glycoforms. The SARS-CoV RBD demonstrated a similar but weaker binding profile, suggesting that this glycan recognition feature may be conserved among lineage B betacoronaviruses [58].

The interaction could facilitate viral attachment in individuals expressing A antigens on airway epithelia (blood group A or AB secretors). In a cellular model, CHO cells expressing ACE2 and engineered to present A-type glycosylation were infected more efficiently by SARS-CoV-2 pseudovirus compared to their O-glycosylated counterparts, despite equivalent ACE2 expression [2]. Although blood group B was not evaluated in this system, other assays reported weaker but detectable RBD binding to the B trisaccharide [2].

**Figure 2:**
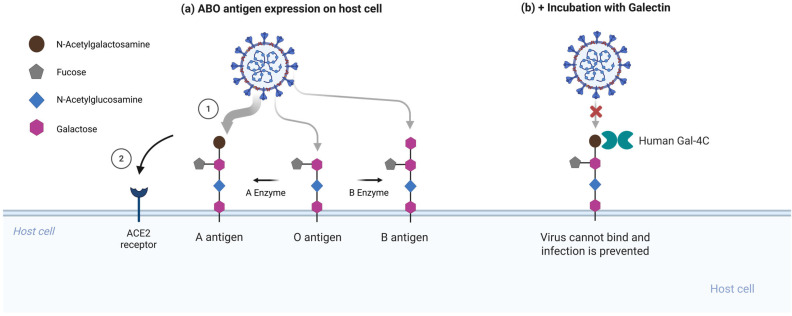
Human galectin-4C inhibits A antigen-dependent enhancement of SARS-CoV-2 entry. (**a**) Enhanced SARS-CoV-2 pseudovirus infection when presenting A-type glycans. The spike RBD preferentially recognizes the blood group A trisaccharide expressed on mucosal epithelial glycoconjugates, with weaker binding to B antigens and unmodified H(O) antigen [2]. (**b**) Galectin-4C, which selectively binds blood group A determinants, inhibits viral entry in A-expressing cells. Galectin-4C hinders glycan attachment sites, reducing virus entry at the epithelial surface. Created in BioRender. Oh, H. (2026) https://biorender.com/w0woy23 (accessed on 3 February 2026).

To test whether RBD binding to A-type glycans contributes to enhanced infection, Stowell and colleagues performed competitive inhibition assays using soluble galectins. Galectin-4C, the C-terminal carbohydrate recognition domain of galectin-4 that preferentially recognizes blood group A determinants, significantly inhibited infection in A-expressing cells, whereas galectin-1, which lacks this preference, had no detectable effect [2]. This supports the conclusion that the viral RBD and galectin-4C compete for the same glycan epitope on host cells [Figure 2].

The blood group A binding capacity of the RBD appears to have been retained across SARS-CoV-2 evolution and may be enhanced in some variants. In binding assays, multiple variants of concern, including Alpha, Delta, and Omicron, maintain reactivity toward blood group A determinants, with Omicron often showing stronger binding than earlier strains [2]. This increase coincides with substantial RBD remodeling in Omicron, raising the possibility that some mutations, while primarily selected for immune escape or enhanced ACE2 affinity, may also potentiate glycan-binding functionality. This could contribute to Omicron’s heightened transmissibility, particularly among individuals with blood group A. Notably, the persistence of ABO-linked susceptibility in epidemiological data throughout Omicron’s global spread supports the continued functional relevance of this interaction [56].

The ABO glycan interaction represents a compelling mechanism by which SARS-CoV-2 subverts host glycosylation for enhanced cell attachment. It bridges population-level clinical observations with molecular-level structural specificity. While the relative contribution of this interaction to transmission and pathogenesis in vivo remains to be investigated, its biochemical existence is unequivocal. These findings highlight the importance of considering host glycan polymorphisms, such as ABO blood type, as determinants of viral infectivity. For a blood group A individual, SARS-CoV-2 may exploit mucosal A antigens on epithelial surfaces (glycolipids or mucin-type O-glycans) to concentrate on the cell surface and facilitate receptor engagement. In contrast, group O individuals, lacking these glycan hooks, may present less favorable adhesion sites, reducing viral retention and the probability of infection.

## 5. Glycan-Mediated Entry Mechanisms in Other Viruses

Understanding how diverse viruses exploit host glycans for attachment, entry, and immune evasion provides a valuable framework to contextualize SARS-CoV-2 glycan interactions. Many pathogenic viruses have evolved lectin-like domains or heavily glycosylated surface proteins that engage carbohydrates (such as sialic acids, glycosaminoglycans, and glycolipids) on host cells. These glycan-mediated interactions can concentrate virions at the cell surface, mediate cell or tissue tropism, and modulate immune recognition. Below, we compare several examples, including the influenza virus, HIV-1, Ebola virus, dengue virus, and human coronaviruses OC43, HKU1, and MERS-CoV, highlighting their glycan-binding mechanisms, structural adaptations, and how these compare to those of SARS-CoV-2 [Table 1].

### 5.1. Human Norovirus (HNoV)

HNoV is a leading cause of acute viral gastroenteritis worldwide and represents one of the best-characterized examples of glycan-dependent viral attachment [87,88,89]. Unlike many enveloped viruses, HNoV is non-enveloped, positive-sense RNA viruses belonging to the family Caliciviridae [88,89]. Their initial attachment to host cells is primarily mediated by interactions with histo-blood group antigens (HBGAs), a diverse group of fucosylated and sialylated glycans expressed on mucosal epithelial surfaces and in bodily secretions [87,90]. Structural and biochemical studies have demonstrated that the protruding (P) domain of the major capsid protein VP1 contains well-defined binding pockets that specifically recognize distinct HBGA motifs, including A, B, H, and Lewis antigens [91,92,93]. These interactions are highly strain-specific and contribute to host susceptibility and population-level resistance patterns. For example, individuals lacking functional FUT2 (non-secretors) do not express certain HBGAs on intestinal epithelia and are largely resistant to infection by many norovirus strains [87,94]. HBGA binding functions as a critical attachment factor that promotes viral concentration at the cell surface and facilitates subsequent engagement with still incompletely defined entry receptors [88,92]. Multivalent interactions between clustered capsid proteins and cell-surface glycans enhance binding avidity, enabling efficient attachment even in the absence of high-affinity protein receptors [88,93]. Recent evidence further suggests that norovirus–glycan interactions may influence viral tropism, immune evasion, and transmission dynamics [89,94]. In addition to HBGAs, some norovirus strains can interact with sialylated glycans and heparan sulfate-like molecules, suggesting a degree of flexibility in glycan usage [97]. Together, these findings highlight HNoV as a paradigmatic model for glycan-mediated viral entry, in which carbohydrate recognition plays a central role in determining host range, infection efficiency, and epidemiological patterns.

### 5.2. Influenza A Virus (IAV)

IAV provides a classic example of glycan-dependent entry. The viral HA is a trimeric surface glycoprotein that functions as a lectin, binding to sialic acid residues on the host cell’s surface glycoproteins and glycolipids [62]. HA-sialic acid binding is multivalent because IAV virions display many HA trimers and each HA protomer carries a receptor-binding site, allowing avid engagement of multiple sialylated receptors on the cell surface, a principle that underlies erythrocyte hemagglutination [18,62]. The binding of HA to sialylated receptor clusters on the cell surface clusters the virus and initiates endocytic uptake [62]. Notably, different IAV strains prefer different sialic acid linkages: avian IAV HA binds α2,3-linked sialic acids, whereas human-adapted HA binds α2,6 linkages [18]. This specificity in glycan receptor linkage and chemistry is a key determinant of IAV host range and tissue tropism [18]. For example, α2,6-linked sialic acids are abundant in the human upper respiratory epithelium, aligning with the sites of human IAV infection, whereas α2,3 linkages are prevalent in avian intestinal tracts, matching the tropism of IAV. Importantly, HA’s engagement of sialic acid itself does not trigger immediate fusion; instead, after endosomal internalization, low pH induces a conformational change in HA that drives membrane fusion and viral entry [62]. The virus carries a second glycoprotein, neuraminidase (NA), which cleaves sialic acids. NA’s receptor-destroying activity frees new virions from decoy receptors and from the host cell upon egress, counteracting HA’s high avidity binding. HA is also glycosylated (typically 5-11 N-linked glycans on the globular head), and these glycans modulate HA function and antigenicity [98,99]. Glycans near HA’s receptor-binding site or cleavage site can reduce receptor affinity or shield HA from proteases, thereby affecting virulence and tropism [100]. Furthermore, variability in HA glycosylation serves as an antigenic drift mechanism: the addition of glycans creates a glycan shield on the viral surface, masking antigenic epitopes from neutralizing antibodies [98,99,101]. This strategy is reminiscent of HIV’s envelope (see below) and, to a lesser extent, SARS-CoV-2’s Spike glycan shielding, though IAV shield is less extensive than HIV’s. In summary, IAV relies on a lectin-glycan interaction (HA-sialic acid) for cell attachment and entry, with multivalency and receptor specificity dictating its host range. It employs a balancing act of receptor binding and receptor destruction (HA vs. NA) to ensure successful infection [18,62].

### 5.3. Human Immunodeficiency Virus (HIV-1)

HIV-1 uses CD4 as its primary receptor and CCR5 or CXCR4 as co-receptors rather than a glycan receptor, yet glycans remain central to attachment and immune evasion [102,103]. HIV-1 gp120, within the Env trimer, is highly N-glycosylated and carries on the order of 25 to 30 N-linked glycans, generating a prominent glycan shield [103,104,105]. These glycans, comprising a mixture of high-mannose and complex-type structures, mask conserved protein epitopes on gp120, thereby enabling escape from many antibody responses [103,106]. Notably, the glycan shield is not simply passive camouflage; it is essential for Env structure and function. For instance, the removal of certain gp120 glycans can disrupt proper folding or diminish binding to CD4, thereby reducing HIV infectivity [104,106]. In fact, the recessed CD4-binding site of gp120 is partially shielded by surrounding glycans, and some broadly neutralizing antibodies (bnAbs) against HIV recognize glycan-dependent epitopes on gp120 [103,106,107,108].

Beyond immune evasion, HIV exploits host glycans for attachment. The envelope’s high-mannose N-glycans are specifically recognized by C-type lectin receptors on host cells. Dendritic cells express DC-SIGN, which binds mannose-rich glycans on gp120 and gp41. This interaction does not immediately neutralize the virus. Instead, it tethers HIV to the dendritic cell surface. HIV can be shuttled to lymph nodes, where dendritic cells trans-infect CD4^+^ T cells by transferring captured virions [66,67]. The interaction between DC-SIGN and gp120, a form of lectin-mediated trans-infection, highlights how multivalent binding of viral glycans by a tetrameric lectin can cluster viruses on immune cells. Likewise, macrophages express the mannose receptor, which can internalize HIV bound via glycans [68]. During mucosal transmission, HIV can also use HSPGs on epithelial and other cells as initial attachment factors. Positively charged regions of gp120, including the V3 loop, can bind negatively charged heparan sulfate chains. Such HSPG-mediated capture increases the local concentration of virus on the cell surface, enhancing the likelihood of Env engaging the true receptor CD4 [69,70,71]. Notably, soluble heparin and other polyanions can inhibit HIV by competing for these HSPG-binding sites on gp120 [47]. In summary, while HIV’s entry is ultimately protein-receptor-mediated, the virus extensively exploits glycans: its own dense glycans form a shield and ligand for lectin receptors, and it binds HSPGs to promote attachment. Glycans facilitate HIV attachment while also contributing to immune evasion, and similar concepts may apply to SARS-CoV-2. SARS-CoV-2 spike is heavily glycosylated, though less densely than gp120, and virions may be captured by lectins or heparan sulfate proteoglycans on host cells [8,103], but the specifics of HIV’s glycan interactions are uniquely refined to its life cycle.

### 5.4. Ebola Virus (EBOV)

EBOV, a filovirus, employs glycan-mediated strategies for both cell attachment and immune evasion. The sole envelope protein, Ebola glycoprotein (GP), is a class I fusion protein functionally analogous to coronavirus Spike or HIV Env [109]. EBOV GP is heavily glycosylated, containing multiple N-linked glycans and an extensive mucin-like domain rich in O-linked glycans [110,111,112]. These sugars form a thick coat on GP, conferring a lectin-binding phenotype and masking underlying protein epitopes [111,112]. Initial attachment of Ebola virions to host cells is mediated, in part, by interactions between the GP and cell-surface glycans. In particular, EBOV can bind heparan sulfate proteoglycans on target cells, a strategy shared by many other viruses [72]. Experimental evidence shows that soluble heparin (a heparan sulfate mimetic) competitively inhibits Ebola virus infection by preventing GP1 from attaching to cells [72]. This implies that cell-surface heparan sulfate is an important attachment factor for Ebola, facilitating the concentration of virions on cellular membranes.

Moreover, the Ebola virus actively targets host lectins for attachment. The dense array of mannose and other sugars on EBOV GP is recognized by C-type lectin receptors on immune cells. Dendritic cell- and liver-expressed lectins, such as DC-SIGN, L-SIGN, and LSECtin, all bind to Ebola GP, enhancing viral attachment and uptake [73,74,75]. DC-SIGN on macrophages and dendritic cells can bind Ebola GP glycans, especially in the mucin-like domain, facilitating virion capture and internalization by antigen-presenting cells [73,76]. Similarly, LSECtin on liver sinusoidal endothelial cells binds to EBOV GP, potentially facilitating liver tropism [75]. These lectin-mediated interactions do not trigger immediate fusion but primarily promote virion capture and uptake, with Ebola virus entering cells predominantly via macropinocytosis [113]. In endolysosomal compartments, host cathepsins proteolytically process GP, removing the glycan-rich mucin-like domain and exposing the receptor-binding core of GP1 [114,115]. This processed GP then engages the endosomal protein NPC1, which is the critical trigger for membrane fusion [116,117]. Thus, in Ebola’s entry, glycans serve as attachment enhancers and immune decoys: the virus uses HSPGs and lectin receptors to adhere to cells, while the bulky glycans on GP shield key epitopes from neutralizing antibodies. In fact, the mucin-like domain contributes to EBOV’s evasion of antibody-mediated neutralization and is thought to be a factor in its extreme pathogenicity [111,112,118]. In comparison to SARS-CoV-2, which also can use cell-surface HSPGs for initial attachment [75], Ebola’s reliance on lectins is more pronounced and is coupled with a dedicated strategy of glycan shielding via its mucin domain. Both viruses highlight the importance of multivalency and receptor clustering. Ebola GP can engage multiple lectins on the cell surface, and models of SARS-CoV-2 entry propose that spike-ACE2 binding can be facilitated by concurrent interactions with HSGPs [72,75].

### 5.5. Dengue Virus (DENV)

DENV, a flavivirus, exemplifies how viruses can utilize both glycoprotein-associated glycans and host cell surface glycans for entry. The dengue virion is enveloped and displays 180 copies of the envelope (E) glycoprotein arranged with icosahedral symmetry [119]. Each E monomer has one or two N-linked glycosylation sites that carry high-mannose or complex glycans. These viral glycans play a crucial role in facilitating dengue virus infection [119,120]. Like many flaviviruses, DENV uses cell-surface glycosaminoglycans, especially heparan sulfate (HS), as an initial attachment factor [77,79]. The E glycoprotein binds to negatively charged HS chains on cell membranes, tethering the virus electrostatically to the cell. This attachment is of relatively low affinity per glycan but is highly multivalent: numerous E proteins on the virion can engage multiple HS chains on the cell surface, leading to high-avidity binding. Consistently, highly sulfated heparin can potently inhibit DENV infection in cell culture by competing with cell-surface HS for E protein binding [78,79]. Mapping studies implicate basic residues on the envelope (E) protein in heparan sulfate binding, including Lys291 and Lys295 in some flaviviruses. Heparin competition at this site can inhibit viral entry [79]. Beyond glycosaminoglycans, DENV can also attach to host lectin receptors. The high-mannose N-glycan on E (Asn153) is a ligand for DC-SIGN on dendritic cells, analogous to HIV’s gp120 mannose patch. Dendritic cells in the skin capture DENV via DC-SIGN, which enhances the infection of these cells and facilitates their dissemination to lymph nodes [80,81]. Indeed, DC-SIGN is known as an attachment receptor for many flaviviruses, and the presence or absence of the E protein glycan can determine a strain’s ability to utilize these lectins [80,81,82]. This highlights how virion glycosylation may influence tropism. Mosquito-derived DENV is often enriched for high-mannose glycans and can be recognized more efficiently by DC-SIGN and mannose-binding lectin (MBL) than mammalian cell-derived virus, which tends to present more processed hybrid and complex-type glycans [121,122].

Dengue virus also demonstrates glycan-related immune evasion strategies, particularly through its secreted non-structural protein 1 (NS1). NS1 is an abundantly secreted glycoprotein (dimer or hexamer) that is heavily glycosylated and can bind to cell surfaces via interactions with HS and chondroitin sulfate E [123]. While not involved in virion entry, NS1 plays a key role in modulating the host immune response. The host MBL, a soluble pattern-recognition molecule of the lectin complement pathway, can bind high-mannose glycans on the DENV virion surface and neutralize the virus by blocking attachment and/or promoting complement deposition [124,125]. DENV counteracts this defense in part through NS1. Secreted NS1 can bind MBL in the circulation, functioning as a decoy that sequesters MBL and reduces its availability for binding to virions [124]. By sequestering MBL, NS1 protects dengue virions from MBL-mediated neutralization and complement activation, thereby enhancing viral survival and spread [124]. This is a striking example of a viral glycoprotein subverting the host’s glycan-binding immune surveillance. In summary, DENV uses glycans in multiple ways, promoting attachment via heparan sulfate and lectins while also antagonizing soluble lectin-mediated immunity. Compared to SARS-CoV-2, which similarly can be inhibited by heparin and whose Spike protein engages HS [24,126], dengue’s reliance on HS is perhaps even more pronounced as a primary attachment mechanism. Both viruses underscore multivalency, in which many weak protein-glycan interactions combine to yield strong overall binding and concentrate virions on target cells prior to receptor engagement [127].

### 5.6. Seasonal Human Coronaviruses

Human betacoronaviruses OC43, HKU1, and alphacoronaviruses NL63, 229E cause seasonal respiratory infections [128]. Notably, HCoV-OC43 and HCoV-HKU1 rely on host sialoglycans as their entry receptors, in contrast to SARS-CoV-2, which primarily uses ACE2. OC43 and HKU1 have long been known to agglutinate red blood cells and to bind to O-acetylated sialic acids on host cells [63,129]. In fact, for these viruses, the glycan is the principal receptor for entry. Both OC43 and HKU1 recognize 9-O-acetylated N-acetylneuraminic acid (Neu5,9Ac2) on glycoproteins or glycolipids of the target cell [36,63]. This specificity extends to defined glycan motifs. Glycan array studies suggest that the HKU1 spike binds most strongly to a disialylated glycolipid bearing 9-O-acetylated, α2,8-linked sialic acid, consistent with a GD3-like motif. In contrast, OC43 shows broader recognition of 9-O-acetyl sialic acids across α2,3, α2,6, and α2,8 linkages [36,64]. Preference for 9-O-acetylated sialosides may contribute to tissue tropism, as these modifications are enriched in the human upper respiratory tract and align with the typical infection sites of OC43 and HKU1 [63]. Consistent with their glycan-dependent entry, these viruses encode a second envelope glycoprotein called hemagglutinin-esterase (HE). It is a sialic acid-binding and receptor-destroying enzyme, conceptually analogous to the influenza A/B receptor-binding and receptor-destroying functions and most closely resembling the influenza C hemagglutinin-esterase-fusion (HEF) protein [129]. HE binds 9-O-acetylated sialic acids and possesses acetyl esterase activity that removes the 9-O-acetyl groups [130,131]. This activity likely removes sialic acids from receptors and mucins in the respiratory tract, facilitating virion release and limiting mucus entrapment, analogous to influenza neuraminidase-mediated egress [130]. Intriguingly, OC43 and HKU1 differ in how they utilize their HE: both have HEs with reduced enzymatic activity against multivalent mucin-presented glycans, an adaptation to avoid prematurely stripping receptors in the mucus before the virus reaches target epithelium [132]. Instead, the viruses likely rely on their Spike protein for initial attachment to cell-surface sialoglycans and use HE to selectively clear off decoy glycans.

Structurally, the Spike proteins of OC43 and HKU1 contain an NTD that serves as the lectin domain for sialic acid binding [64,65]. High-resolution studies using cryo-EM and crystallography have identified a conserved pocket in the Spike NTD of these embecoviruses that accommodates the 9-O-acetylated sialic acid [65,130]. Key residues line this pocket to contact the acetyl moiety and the sialic acid’s carboxyl and glycerol groups [64,133]. The binding affinity of monomeric NTD for its glycan ligand is moderate (in the low micromolar KD range), but, as with influenza, the virion displays multiple spikes allowing multivalent avidity to reach effective high-affinity attachment [65,132]. OC43/HKU1 Spike engagement with sialylated glycan receptors is sufficient to mediate viral entry via endocytosis. Unlike SARS-CoV and SARS-CoV-2, HCoV-OC43 uses 9-O-acetylated sialic acids as its primary receptor determinant, with infection proceeding via endocytic uptake rather than a defined protein receptor interaction [63,134]. By contrast, SARS-CoV-2’s Spike NTD does not have a strong sialic acid-binding activity, and SARS-CoV-2 requires the protein receptor ACE2 for entry [36,63]. The carbohydrate-binding specificity of these seasonal coronaviruses also influences their host range: OC43 is thought to have originated from a zoonotic transmission of a bovine coronavirus that also binds 9-O-acetylated sialoglycans [129,135,136]. Adaptation to humans may have tuned the Spike’s specificity and the HE’s activity to match human airway glycan patterns [137,138]. In summary, HCoV-OC43 and HKU1 illustrate a strategy where a coronavirus Spike behaves like a lectin, much like influenza’s HA. They rely on sialylated glycan receptors for attachment and entry, utilizing a conserved spike NTD pocket to bind 9-O-acetylneuraminic acid [36,63,64]. They also possess a receptor-destroying enzyme (HE) to refine their interactions with host glycans [130]. These features are a point of contrast with SARS-CoV-2, which lacks an HE and whose Spike has only a weak, possibly incidental sialic acid affinity. Nonetheless, it is notable that close relatives of SARS-CoV-2 within the betacoronavirus genus include lineages with well-established glycan-binding receptor usage, highlighting that coronaviruses can employ either protein receptors or glycan determinants in a lineage-specific manner.

Human alphacoronaviruses NL63 and 229E also employ glycan-dependent attachment mechanisms that complement their primary protein receptor usage [95,139]. HCoV-NL63 utilizes angiotensin-converting enzyme 2 (ACE2) as its main entry receptor, similar to SARS-CoV-2.; however, viral attachment is markedly enhanced by interactions with heparan sulfate proteoglycans on the host cell surface [95]. These negatively charged polysaccharides function as auxiliary attachment factors that promote virion accumulation at the plasma membrane and facilitate subsequent ACE2 engagement. Consistently, enzymatic removal or competitive inhibition of heparan sulfate significantly impairs NL63 attachment and infectivity, underscoring the functional importance of glycan-mediated adhesion during early stages of infection [95,96].

In contrast, HCoV-229E primarily employs human aminopeptidase N (hAPN/CD13) as its entry receptor [140]. Nevertheless, prior to high-affinity receptor binding, 229E virions can interact with cell-surface glycans, including sialylated and sulfated glycoconjugates, which facilitate initial viral docking [63,83]. These low-affinity, reversible interactions increase the local concentration of virions at receptor-rich membrane regions, thereby enhancing the efficiency of productive hAPN engagement. Such glycan-assisted attachment may be particularly important in the respiratory epithelium, where receptor availability and mucosal barriers can limit direct virion–receptor encounters [63].

Collectively, NL63 and 229E illustrate a hybrid entry strategy in which glycan-mediated attachment cooperates with protein receptor recognition to optimize entry efficiency and modulate cellular tropism [137,141]. Unlike OC43 and HKU1, which rely primarily on sialylated glycans as bona fide entry receptors, these alphacoronaviruses utilize host glycans predominantly as auxiliary attachment factors that enhance receptor accessibility and stabilize virion–cell interactions. This distinction highlights the evolutionary flexibility of coronaviruses in exploiting host carbohydrate structures and reinforces the conserved role of glycans as critical determinants of coronavirus attachment and infection across diverse lineages [137,141].

### 5.7. Middle East Respiratory Syndrome Coronavirus (MERS-CoV)

MERS-CoV is a pathogenic betacoronavirus that caused outbreaks of severe respiratory disease from 2012 to 2015. In terms of receptor usage, MERS-CoV is intermediate between the strictly glycan-dependent OC43/HKU1 and the strictly protein-dependent SARS-CoV-2. MERS-CoV employs a dual-receptor strategy: it recognizes a protein receptor (dipeptidyl peptidase 4, DPP4) for entry, but it also has a glycan-binding capacity that contributes to attachment [142,143]. Specifically, the MERS-CoV Spike N-terminal domain (S1-NTD) binds to sialylated glycans on host cells [84,85]. Studies indicate that MERS-CoV spike can attach to α2,3-linked sialic acids on airway epithelial cells, which may promote initial adhesion and increase the likelihood of subsequent receptor engagement [85]. For example, ciliated cells in the human airway are enriched for α2,3-sialylated glycans that can support MERS-CoV attachment [86]. However, this interaction is not sufficient for entry. Productive infection requires the engagement of the MERS-CoV receptor-binding domain with DPP4, along with protease-dependent activation of the spike to enable membrane fusion [142,143,144]. Thus, MERS-CoV’s glycan binding is more of an attachment factor role, analogous to a virus using heparan sulfate or sialic acid to boost infectivity. Consistent with this, pretreatment of cells with sialidase or competition with soluble sialic analogs reduces MERS-CoV infection, but not as completely as blocking DPP4 [84]. In structural terms, MERS-CoV’s sialic acid-binding site in the NTD is defined by specific loops, similar to OC43/HKU1, though with different residue composition; it binds sialosides only weakly (micromolar affinity) compared to OC43/HKU1, indicating it is likely an auxiliary interaction [84]. Indeed, this glycan-binding feature is not uniformly conserved in all MERS-related viruses, suggesting it is an adaptation of MERS-CoV that may enhance infection of certain cell types [84,144]. The presence of both glycan and protein receptors endows MERS-CoV with a form of receptor tropism plasticity: the virus can initially attach broadly via ubiquitous sialylated glycans, potentially facilitating the crossing of mucus or the infection of intermediate cell targets, and then specifically bind to DPP4 on susceptible cells for entry [84,144]. Notably, this dual-receptor strategy parallels the entry of some paramyxoviruses. Measles uses a protein receptor, but whose hemagglutinin can bind SLAM or nectin as well as display lectin activity [84,144]. It also draws an interesting comparison to SARS-CoV-2: while SARS-CoV-2 does not appear to have a functional sialic-acid-binding site in its NTD, both viruses (MERS and SARS-CoV-2) rely on a protein receptor for triggering fusion (DPP4 and ACE2, respectively) and both can be aided by attachment to cell-surface glycans (sialic acid or HS) [24,145]. Additionally, like SARS-CoV-2, the MERS-CoV Spike is extensively glycosylated (covering ~20 N-linked sites), which forms a partial shield. The MERS-CoV glycan shield is generally less dense than that of HIV Env, and it may be less directly involved in regulating receptor accessibility than in SARS-CoV-2, where specific N-glycans can influence RBD exposure and immune recognition [146]. Nonetheless, MERS-CoV glycosylation likely contributes to proper folding and to partial shielding from antibody recognition [21,146].

In summary, MERS-CoV highlights a hybrid mechanism: its Spike NTD has a lectin-like function (binding sialylated glycan receptors), while its Spike CTD engages a protein receptor (DPP4) to mediate entry [84,85]. Glycan binding by MERS-CoV enhances virion attachment and may shape the earliest steps of cellular targeting, favoring cells enriched in the relevant α2,3-sialylated glycans, such as subsets of airway epithelial cells. In contrast, engagement of the protein receptor DPP4 is the primary determinant of productive tropism and, together with protease activation, enables membrane fusion. This two-step mechanism is conceptually similar to how SARS-CoV-2 utilizes cell-surface HSPGs in conjunction with ACE2 [24]. However, SARS-CoV-2’s NTD does not appear to be a specialized lectin domain as in MERS; instead, SARS-CoV-2 relies on heparan sulfate and possibly other cofactors in a more promiscuous manner for initial contact.

### 5.8. Similarities and Differences with SARS-CoV-2

A unifying theme is that many viruses exploit multivalent, low-affinity glycan binding to enhance their attachment to host cells. SARS-CoV-2 is no exception. Although its primary receptor is ACE2, it has been demonstrated that SARS-CoV-2 Spike also binds to heparan sulfate on the cell surface, and this interaction facilitates virus attachment and infection [24]. This is analogous to HIV and Dengue virus using HSPGs, or MERS-CoV using sialylated glycans as auxiliaries to concentrate virions at the cell surface [77,147,148]. In terms of lectin-like domains, SARS-CoV-2’s Spike NTD does not have a well-defined glycan-binding pocket like influenza HA or OC43 Spike. There is some evidence that SARS-CoV-2 NTD can bind sialylated glycolipids (gangliosides) or sialic acid in silico and in vitro, but these interactions are relatively weak and not conserved across all sarbecoviruses [64,146]. Thus, compared to HCoV-OC43/HKU1 or influenza (where glycan binding is the central receptor interaction), SARS-CoV-2′s glycan interactions are more ancillary. On the other hand, SARS-CoV-2′s reliance on ACE2 means it aligns with viruses like HIV and MERS, which require a specific protein receptor to trigger entry [107,147].

A key similarity across these viruses is the concept of a glycan shield. SARS-CoV-2 Spike is decorated with ~22 N-linked glycans that cover a substantial portion of its surface area, though not as densely or uniformly as HIV-1 gp120 [21]. These glycans on the SARS-CoV-2 Spike protein mask immunogenic epitopes, contributing to immune evasion, much like the glycans on HIV or Ebola GP [107,108,146]. However, the SARS-CoV-2 glycan shield is incomplete. The virus exposes certain protein surfaces necessary for ACE2 binding, and indeed, some potent neutralizing antibodies manage to circumvent the glycans [145]. In contrast, HIV’s shield is more encompassing, making it notoriously difficult for the immune system to target [107].

In terms of glycan-mediated tropism, SARS-CoV-2 does not exhibit the stark glycan linkage specificity seen in influenza or OC43. It does not discriminate between cell types based on sialic acid linkages, for instance. Instead, SARS-CoV-2′s tropism is largely governed by ACE2 expression and protease availability. Nevertheless, the ubiquity of HS on human cells means SARS-CoV-2 potentially attaches to a wide variety of cell types, but productive infection still requires ACE2. This contrasts with OC43/HKU1, where the presence of 9-O-acetylated sialic acids on a cell is a major determinant of whether that cell can be infected [63]. Influenza tissue tropism within the respiratory tract is strongly influenced by the distribution of α2,6- versus α2,3-linked sialic acids [36,64]. By contrast, no comparably well-established glycan linkage-based filter has been defined for SARS-CoV-2. One interesting parallel is the role of receptor clustering and conformational priming [24]. For influenza, HA-sialic acid binding clusters virions and can promote uptake. For SARS-CoV-2, binding to HSPGs may cluster Spikes and present them optimally to ACE2 receptors, possibly even inducing conformational changes that favor RBD exposure or furin site cleavage (some studies suggest that glycan engagement might modulate Spike conformation) [24,146]. Similarly, in MERS-CoV, attachment to sialylated glycans may increase spike residence time and promote a receptor-competent orientation that facilitates subsequent DPP4 engagement. DPP4 binding, together with protease-dependent activation, then drives conformational changes that enable fusion [85,147]. This sequential attachment-then-receptor engagement paradigm parallels proposed roles for heparan sulfate in enhancing ACE2-dependent entry of SARS-CoV-2 [24].

Finally, the viruses discussed highlight different balances between glycan dependence and the use of protein receptors. SARS-CoV-2 sits closer to the HIV/MERS end of the spectrum rather than the influenza/OC43 end. Across these viruses, SARS-CoV-2 likewise interfaces with host glycobiology. Spike is extensively glycosylated and has been reported to interact with lectins such as DC-SIGN and L-SIGN. It is inhibited in vitro by soluble polyanionic competitors of glycosaminoglycan binding, including heparin and suramin [24,66,121]. As research progresses, additional parallels are being explored. Recent reports suggest that certain human lectins, such as Siglecs and galectins, may interact with SARS-CoV-2 glycan structures and influence immune responses, following a principle similar to that of lectin-mediated effects described for influenza and HIV [107,108].

In conclusion, comparison of glycan-mediated entry mechanisms across viruses reveals recurring principles: multivalency, in which many weak interactions sum to high-avidity attachment; lectin-glycan specificity, which shapes tropism and host range; the balance between receptor avidity and accessibility, which enables stable binding while permitting receptor release or fusion triggering; and glycan-driven immune evasion, including shielding and decoy strategies. SARS-CoV-2, while primarily dependent on ACE2, still leverages these glycan-based tactics to a significant extent. Its use of heparan sulfate attachment and a partially glycosylation-cloaked Spike reflects a convergence of strategies seen in the above viruses, underscoring the importance of the glycan web at the virus–host interface in viral infection and pathogenesis [21,24].

## 6. Host Lectin-Mediated Protective Outcomes

While viruses frequently exploit host C-type lectin receptors (CLRs) to facilitate entry and dissemination, these receptors primarily function as immune sentinels. The host utilizes interactions with specific CLRs, such as the mannose receptor (MR), CLEC9A, and DCIR, to mount effective antiviral defenses, enhance antigen presentation, and regulate potentially damaging inflammation.

The macrophage mannose receptor (MR, CD206) functions as a pattern recognition receptor (PRR) that binds terminal mannose, fucose, or GlcNAc residues on viral glycoproteins. In the context of SARS-CoV-2, MR captures the spike glycoprotein via its high-mannose N-glycans [50]. Unlike ACE2-mediated entry, this uptake is generally non-productive and routes virions to endolysosomal compartments for degradation [50]. This process facilitates antigen presentation, allowing macrophages and dendritic cells to prime T cells and initiate robust antiviral responses early in infection [51].

Beyond viral capture, specific lectin interactions are crucial for activating CD8^+^ T cell responses, which are essential for clearing intracellular pathogens. CLEC9A (DNGR-1), a receptor expressed on specific dendritic cell subsets (cDC1s), plays a key role in this process. Interactions between CLEC9A and ligands associated with vaccinia virus (VACV) and herpes simplex virus (HSV) infection facilitate the cross-presentation of viral antigens [149,150]. This binding enhances the efficiency of antigen delivery to cytotoxic T cells, thereby accelerating viral clearance and contributing to host recovery [149].

Protective outcomes are not limited to viral elimination but also involve the limitation of immunopathology. The dendritic cell immunoreceptor (DCIR) serves as an important regulatory node during viral infections. For example, interactions between chikungunya virus (CHIKV) and DCIR trigger inhibitory signaling pathways that modulate host immune responses [151]. By dampening excessive pro-inflammatory signaling, DCIR-mediated regulation helps reduce pathological immune reactions and prevents collateral tissue damage during antiviral defense [151].

Collectively, these examples involving MR, CLEC9A, and DCIR illustrate that the host glycan network is not merely a vulnerability exploited by viruses. Rather, it represents a dynamic immunological interface in which lectin-mediated recognition promotes antigen presentation and immune regulation, thereby balancing viral clearance with tissue protection.

## 7. Conclusions

The study of glycan interactions in SARS-CoV-2 entry has shed light on a sophisticated layer of virus–host interplay, transforming our understanding of host glycans from passive structural components to dynamic participants in the infection process. Far from serving merely as a sugar shield, glycans facilitate viral attachment, promote proximity to entry receptors, and in some cases act as co-receptors themselves. SARS-CoV-2 capitalizes on this complexity: its Spike protein is not only a fusogen and ACE2 binder but also a lectin-like glycan sensor, tuned to recognize specific host carbohydrate motifs. The RBD engages blood group A trisaccharides and heparan sulfate; the NTD reveals affinity for sialylated glycans; and the virus’s own glycan coat becomes a substrate for host lectins like galectins and Siglecs.

This glycan-binding repertoire offers several advantages. Attachment to ubiquitous glycans, such as sialic acid and heparan sulfate, enhances initial capture in the mucosal environment, thereby raising the local virion concentration at epithelial surfaces. In tissues with low ACE2 expression, these interactions may facilitate viral entry into receptor-rich zones. Specificity for ABO(H) antigens and sialylated gangliosides could further tune tissue tropism and explain inter-individual variation in susceptibility, as suggested by epidemiological correlations with blood group and variant-dependent binding enhancements.

From a structural and mechanistic perspective, these interactions reflect a highly adaptive strategy. Glycan contacts are often low-affinity individually but collectively confer avidity through multivalency. The Spike’s glycosylation pattern, considered primarily a shield, also contributes functionally, offering ligands for host lectins or stabilizing Spike conformation. Meanwhile, host lectins such as galectin-3, galectin-9, and Siglec-1 can paradoxically aid the virus, clustering virions at the cell surface or shuttling them between immune cells, as observed in trans-infection phenomena.

The functional role of glycan interactions in coronavirus entry presents a valuable opportunity for translational applications. Coronaviruses such as SARS-CoV-2 and the endemic HCoVs (OC43, HKU1, NL63, 229E) engage host cell-surface glycans (including heparan sulfate and sialylated glycans) for viral capture, adhesion, and receptor engagement. These interactions, often mediated by the N-terminal domain of the Spike protein, can be therapeutically targeted to interrupt the earliest stages of infection.

Several classes of glycan-based therapeutics have emerged from this paradigm. Heparin derivatives and non-anticoagulant heparan sulfate mimetics, such as pixatimod and HTCC, have been shown to inhibit SARS-CoV-2 binding and entry by competing with host heparan sulfate for Spike interaction. Similarly, sialic acid analogs and sialylated decoys are being explored to block OC43/HKU1 Spike recognition. These approaches offer the advantage of targeting relatively conserved host molecules, reducing the likelihood of viral resistance.

Additionally, glycan-binding lectins such as griffithsin have demonstrated potent antiviral activity by cross-linking viral glycans and preventing conformational changes required for membrane fusion. Diagnostic applications are also under development, including glycan-coated biosensors and lectin-functionalized nanoparticles, which exploit these interactions for virus detection and characterization.

Targeting glycan-mediated attachment represents a complementary strategy to protein-focused antivirals, with potential utility across multiple coronavirus lineages. As our molecular understanding of the glycan interactome deepens, so too will our capacity to design broad-spectrum inhibitors and personalized antiviral interventions based on glycan diversity and distribution.

In summary, SARS-CoV-2 reveals a modern paradigm of glycan-mediated entry: not a redundant add-on to ACE2 usage, but a nuanced, evolutionarily conserved strategy that enhances infectivity, modulates host range, and intersects with innate immune recognition. Understanding this glycan web will be crucial not only for addressing current challenges but also for anticipating the entry tactics of future emergent viruses.

## Figures and Tables

**Table 1:** Glycan-Mediated Entry Mechanisms of Viruses.

Virus	Viral Protein/ Component	Host Glycan/Receptor/Lectin	Functional Outcome	References
Influenza Virus	Hemagglutinin (HA)	Sialic Acid (α2,3 or α2,6 linkages)	Primary Receptor: Mediates attachment and determines host tropism (avian vs. human).	[18,62]
HCoV-OC43/ HKU1	Spike (NTD)	9-O-Acetylated Sialic Acids	Primary Receptor: Essential for attachment and entry (sialic acid serves as the main receptor).	[36,63,64,65]
HIV-1	gp120 (V3 loop)	Heparan Sulfate Proteoglycans (HSPGs) DC-SIGN (Dendritic Cells)/Mannose Receptor (Macrophages)	Attachment: Electrostatic interaction concentrates virions on cell surfaces to facilitate CD4 engagement. Trans-infection: Tethers virions to immune cells for transport to lymph nodes and transfer to T cells.	[66,67,68,69,70,71]
Ebola Virus (EBOV)	Glycoprotein (GP)	Heparan Sulfate Proteoglycans (HSPGs) DC-SIGN/L-SIGN/LSECtin	Attachment: Enhances virion concentration on the cell membrane. Uptake & Entry: Promotes capture by antigen-presenting cells and liver sinusoidal endothelial cells.	[72,73,74,75,76]
Dengue Virus (DENV)	Envelope (E) Protein	Heparan Sulfate (HS) DC-SIGN Mannose-Binding Lectin (MBL)	Attachment: Basic residues on E protein bind negatively charged HS for initial tethering. Cellular Tropism: Facilitates infection of dendritic cells. Host Defense: Soluble MBL binds virions to neutralize infection or trigger complement.	[77,78,79,80,81,82]
HCoV-229E	Spike	Sialylated/Sulfated Glycans	Auxiliary Attachment: Facilitates initial docking before binding to hAPN (CD13).	[63,83]
MERS-CoV	Spike (NTD)	α2,3-linked Sialic Acids	Auxiliary Attachment: Promotes adhesion to airway epithelium; entry requires DPP4.	[84,85,86]
Human Norovirus	VP1 (Capsid P domain)	Histo-Blood Group Antigens (HBGAs) (A, B, H, Lewis)	Primary Attachment: Concentrates virions at mucosal surfaces; confers strain-specific host susceptibility (e.g., secretor status).	[87,88,89,90,91,92,93,94]
HCoV-NL63	Spike	Heparan Sulfate Proteoglycans (HSPGs)	Auxiliary Attachment: Enhances infection; primary entry is mediated by ACE2.	[95,96]

## Data Availability

No new data were created or analyzed in this study. Data sharing is not applicable to this article.

## Abbreviations

The following abbreviations are used in this manuscript:

ACE2	Angiotensin-Converting Enzyme 2
APC	Antigen-Presenting Cell
CHIKV	Chikungunya Virus
CLEC9A	C-type Lectin Domain Family 9 Member A (DNGR-1)
CLR	C-type Lectin Receptor
COVID-19	Coronavirus Disease 2019
CRD	Carbohydrate Recognition Domain
Cryo-EM	Cryogenic Electron Microscopy
DC	Dendritic Cell
DCIR	Dendritic Cell Immunoreceptor
DC-SIGN	Dendritic Cell-Specific Intercellular adhesion molecule-3-Grabbing Non-integrin (CD209)
DENV	Dengue Virus
DPP4	Dipeptidyl Peptidase-4
EBOV	Ebola Virus
FUT2	Fucosyltransferase 2
Gal	Galectin (e.g., Gal-3, Gal-9)
GlcNAc	N-Acetylglucosamine
GM1/2/3	Ganglioside Monosialic Acid 1, 2, 3
gp120	Glycoprotein 120 (HIV)
HA	Hemagglutinin
HBGA	Histo-Blood Group Antigen
HCoV	Human Coronavirus
HE	Hemagglutinin-Esterase
HIV-1	Human Immunodeficiency Virus Type 1
HS	Heparan Sulfate
HSPG	Heparan Sulfate Proteoglycan
HSV	Herpes Simplex Virus
LacNAc	N-Acetyllactosamine
L-SIGN	Liver/Lymph Node–Specific Intercellular adhesion molecule-3-Grabbing Integrin
MBL	Mannose-Binding Lectin
MERS-CoV	Middle East Respiratory Syndrome Coronavirus
MGL	Macrophage Galactose-Type Lectin
MR	Mannose Receptor (CD206)
NA	Neuraminidase
Neu5Ac	N-Acetylneuraminic Acid (Sialic Acid)
NK	Natural Killer (Cell)
NMR	Nuclear Magnetic Resonance
NS1	Non-Structural Protein 1
NTD	N-Terminal Domain (of Spike)
RBD	Receptor Binding Domain (of Spike)
SARS-CoV-2	Severe Acute Respiratory Syndrome Coronavirus 2
Siglec	Sialic Acid–Binding Immunoglobulin-Type Lectin
S1/S2	Spike Protein Subunits 1 and 2
TIM-3	T-cell Immunoglobulin and Mucin-Domain Containing-3
TMPRSS2	Transmembrane Serine Protease 2
VACV	Vaccinia Virus
